# Epithelioid Variant of Pleomorphic Liposarcoma: A Rare Challenging Diagnosis Causing Severe Medial Thigh Pain

**DOI:** 10.7759/cureus.19531

**Published:** 2021-11-13

**Authors:** Hritik Nautiyal, Abdullah Egiz, Sarvin Farajzadeh Asl, Abdurrahman H Fazail, Sanjana Nautiyal

**Affiliations:** 1 Surgery, University of Central Lancashire School of Medicine, Preston, GBR; 2 Surgery, Royal Blackburn Teaching Hospital, East Lancashire Hospitals NHS Trust, Blackburn, GBR; 3 Radiology, University of Central Lancashire School of Medicine, Preston, GBR; 4 Pathology, Combined Medical Institute, Dehradun, IND

**Keywords:** aggressive sarcoma, surgery, plastic and reconstructive surgery, soft tissue tumours, pleomorphic liposarcoma

## Abstract

We present the case of pleiomorphic liposarcoma in the medial compartment of the thigh of a 59-year-old female patient. The lump was noticed eight months prior to presentation and had gradually increased in size, leading to pressure symptoms in her thigh. Although painless initially, the lump eventually became tender prompting her to seek surgical attention. At the plastic surgery clinic, she was advised to get an MRI, which revealed an irregular but well-defined mass lesion measuring 8.2 x 6.6 x 4.3 cm. The mass did not have any manifestations in the surrounding structures. A wide excisional biopsy was then performed, and multiple sections were processed for histopathological analysis, confirming a diagnosis of epithelioid variant of pleomorphic liposarcoma.

## Introduction

Liposarcoma is the most common malignant soft tissue tumour in adults. They are mesenchymal in origin and are usually large, occurring most frequently in the lower extremities (popliteal fossa and medial thigh), retroperitoneal, perineal and mesenteric region and shoulder area [1]. The common morphological denominator of liposarcoma is the lipoblasts [1,2]. These appear as mono or multinucleated cells with mono or multi-cytoplasmic vacuoles that contain lipids [3]. Liposarcoma can be differentiated into four subtypes: (1) Atypical lipomatous well-differentiated liposarcoma (ALT/WDLS); (2) Dedifferentiated liposarcoma (DLS); (3) Myxoid liposarcoma (MLS) and (4) Pleomorphic liposarcoma (PLS) [4]. PLS is the rarest subtype as it comprises only 5-10% of all lipomatous tumours. It typically occurs in adults aged 50 or above and mostly arises in the deep soft tissue of extremities more than retroperitoneal. PLS also has the highest LS tumour-associated mortality occurring up to 50% of patients. It also has the highest malignancy grade, tissue-invasion, metastatic nature and recurrence rate [4]. Complete surgical resection is challenging but at present, the most successful and only recognised treatment for PLS. Unresectable tumours are managed with radio and chemotherapy, which reduce recurrence but have shown to have no effect on mortality [5]. In addition, histopathology determines the prognostic implications for all lipomatous tumours [1,4,6]. Herein, we present the case of a 59-year-old female with an LPS in her right medial thigh with hepatic, skeletal and pulmonary metastasis. There is limited reporting of such cases in medical literature, highlighting the importance of further research to improve the diagnosis and management of PLS, especially the cases associated with distant metastases.

## Case presentation

A 59-year-old female patient presented to a plastic surgery clinic with a lump on her right medial thigh. She noticed the lump eight months ago and it was gradually increasing in size. It was painless initially; however, the lump gradually became tender. The patient was otherwise fit and well with no co-existing morbidities. Closer examination of the right thigh revealed a high consistency mass, with reduced mobility. There was no right inguinal lymphadenopathy or lymph nodes enlargement. 

**Figure 1:**
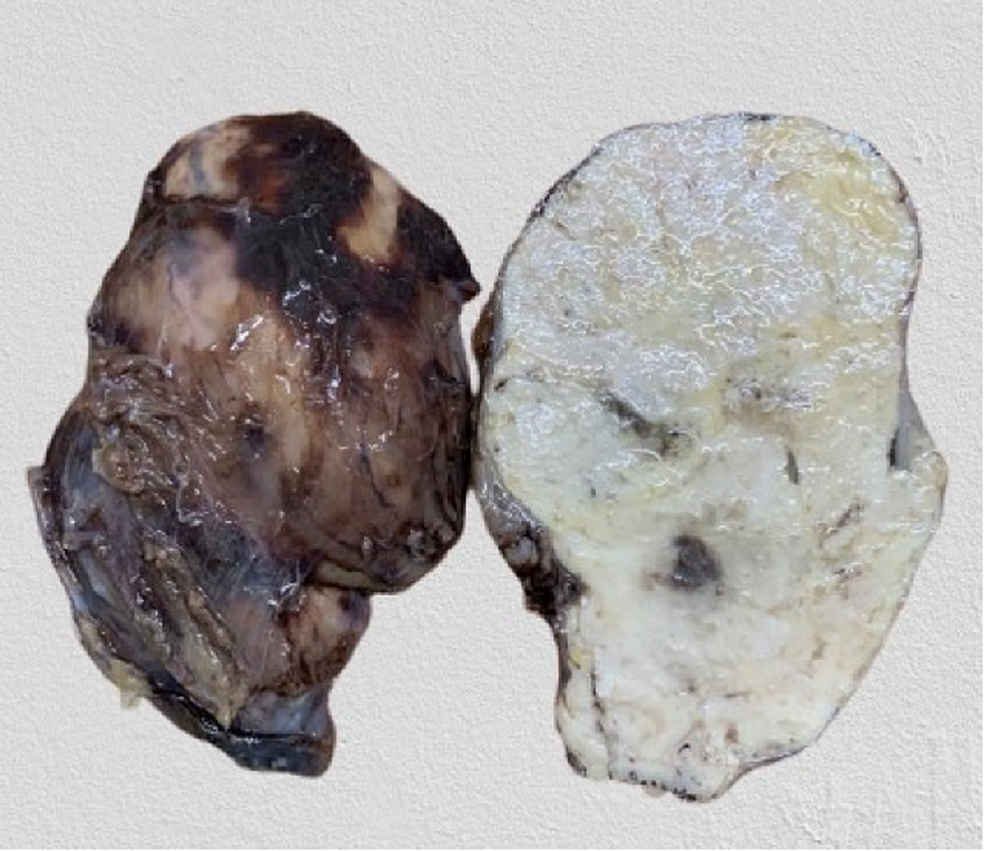
Wide excisional biopsy revealed a smooth mass with soft consistency, measuring 8.5 cm x 6.5 cm x 5.4 cm

Laboratory blood tests were unremarkable. An MRI scan was requested to further assess the mass, which was irregular but well-defined in the medial compartment of the thigh, measuring 8.2 x 6.6 x 4.3 cm in size. The mass showed an intermediate signal on both T1 and T2-weighted imaging, appearing hyperintense compared to the adjacent muscular tissue, which also persisted on fat-suppressed imaging. The mass invaded the intramuscular fat planes and caused compression and displacement of the adductor muscles. There was no evidence of intra-muscular invasion. Normal cortical outline and medullary signal intensity of the femur were seen in the right thigh. There was no evidence of bone contusion, marrow oedema, fracture line, or cortical discontinuity. Other muscles of the thigh had a normal outline and signal intensity with no evidence of focal or diffuse oedema. Neurovascular structures were unremarkable. Overall, MRI findings were suggestive of soft tissue neoplasm, with a possibility of a neurogenic tumour. A wide excisional biopsy was then performed to further assess the tissue histologically by the pathologist and confirm the diagnosis. The removed mass measuring 8.5 x 6.5 x 5.4 cm and is shown in Figure 1. The mass had a smooth outer surface and a soft consistency. The cut surface showed a homogenous tan-white appearance with some slit-like spaces and occasional haemorrhagic spots. Multiple sections were processed for histopathological examination from different planes.

**Figure 2:**
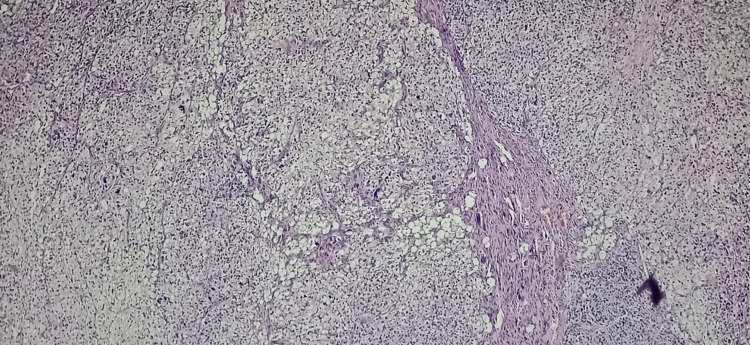
The section shows sheets of adipocytes in low power (10x) – Hematoxylin and Eosin stain (H&E)

**Figure 3:**
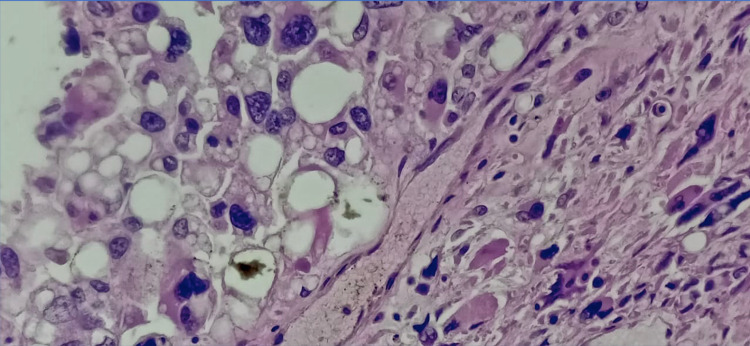
High power magnification (40x) showing numerous lipoblasts with severe nuclear atypia/ bizarre cells and abnormal mitotic figures – Hematoxylin and Eosin stain (H&E)

**Figure 4:**
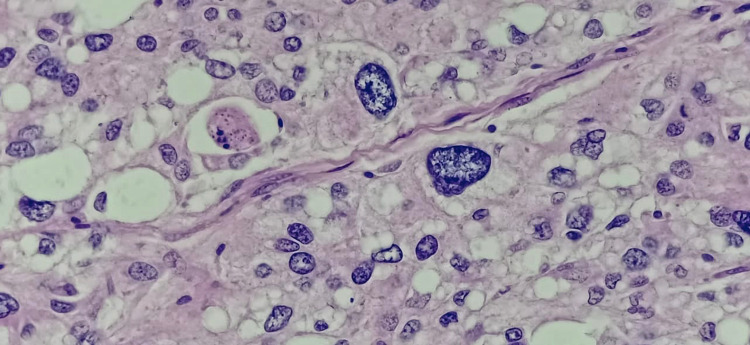
High power magnification (40x) showing numerous lipoblasts with severe nuclear atypia/ bizarre cells and abnormal mitotic figures – Hematoxilin and Eosin stain (H&E)

**Figure 5:**
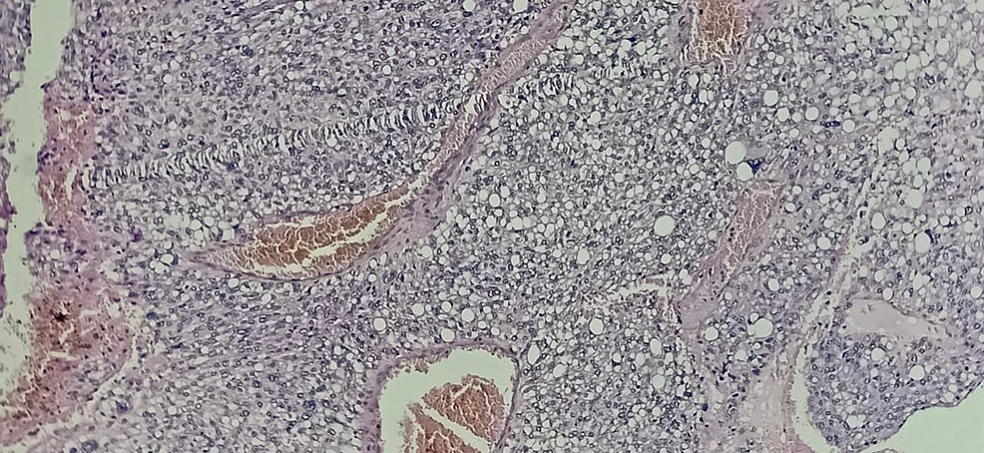
The section shows dilated and congested blood vessels – Hematoxylin and Eosin stain (H&E)

Histopathology of the mass revealed a tumour arranged in sheets and fascicles composed of round to oval cells along with variably sized adipocytes (Figure 2). Numerous interspersed lipoblasts with indented nuclei were also seen. Moreover, severe nuclear atypia was noted including bizarre cells (Figure 3 and Figure 4). An area of necrosis was seen with a few areas, which showed malignant and fibrous histiocytoma-like features. Furthermore, a few thin-walled dilated and congested blood vessels were also noted. Also, brisk mitosis was noted (35-38 per 10 HPF) (Figure 5). Immunohistochemical staining (IHC) was also performed and the tumour cells were diffusely positive for vimentin and focally positive for S-100; while negative for creatine kinase (CK), smooth muscle antigen (SMA), desmin, CD34 and MyoD1. Overall, the pathological findings are all suggestive features of a high-grade sarcoma, favouring the epithelioid variant of PLS.

The patient was also referred to the Department of Nuclear Medicine & Molecular Imaging for post-surgical restaging and assessment for possible metastases using 18F-fluorodeoxyglucose positron emission tomography-computed tomography (18F-FDG PET-CT) scan. The abdominopelvic section showed the liver measuring within normal limits; however, the scan revealed an ill-defined hypermetabolic hypodense lesion seen involving segment VI measuring approximately 2.8 x 1.8 cm suggestive of hepatic metastases with concurrent fatty liver. The scan was also remarkable for the musculoskeletal system, revealing multiple hypermetabolic osseous lesions involving the skull base, right iliac bone, and few vertebrae (predominantly T7-L1); which was suggestive of skeletal metastases. A non-hypermetabolic pulmonary nodule in the right lung field was also detected; it was considered to be likely benign, but interval scanning is suggested to monitor further progress. Finally, there was also evidence of diffuse subtle hypermetabolism at the site of surgery which is suggestive of postsurgical inflammatory sequelae; however, microscopic residual disease cannot be completely ruled out. These multi-organ lesions were due to be biopsied to examine their immunohistochemical findings to confirm metastatic lesions of primary pleiomorphic liposarcoma. However, the patient did not attend the follow-up appointments.

## Discussion

PLS represents the rarest type of liposarcomas [7]. Due to the dearth of literature in the area, diagnosis and treatment of PLS are still significant challenges for clinicians [7,8]. The single most important feature in the diagnosis of PLS is the presence of pleomorphic lipoblasts, the proportion of which varies significantly among cases [9]. That being said, diagnosis of PLS comes with many closely related differentials such as DLS, pleomorphic leiomyosarcoma, pleiomorphic rhabdomyosarcoma, pleomorphic malignant peripheral nerve sheath tumour, and malignant fibrous histiocytoma [8-10]. Although some immunohistochemical markers and ultrastructural evaluation can be used to exclude the alternative diagnoses, careful attention to microscopic detail and the recognition of pleomorphic lipoblasts are the major determinants of the diagnosis [9-12]. 

Several differential diagnoses were excluded following our IHC. Pleomorphic lipoma, for instance, which usually presents as a well-circumscribed mass shows strong immunoreactivity to CD34 (reported negative in this case). Furthermore, other closely resembling carcinomas such as renal cell carcinomas were excluded owing to the negative epithelial membrane antigen test. MFH and other pleomorphic non-lipogenic sarcomas were excluded based on the presence of lipoblasts in this case. Furthermore, as this case demonstrated a significant degree of pleomorphism, myxoid liposarcoma was safely excluded. DLS is another closely related differential; however, the abundance of lipoblasts associated with bizarre nuclei and irregular nuclear membranes pointed towards PLS. Furthermore, the location of the lesion in the extremities is far more common in PLS than in DDLPS, which is mostly found retroperitoneally [8]. As reported in this case, the epithelioid variant of PLS, which represents only around 10% of all PLS, is characterized by a sheet-like arrangement of epithelioid cells with abundant eosinophilic cytoplasm. Less obvious adipocytic differentiation and focal positivity for S-100 increases potential for misdiagnosis as carcinoma or melanoma, further highlighting the importance of the pathologist’s attention to detail for an accurate diagnosis. 

The mainstay of treatment for soft tissue sarcomas remains complete surgical resection to minimize the risk of recurrence [5,13-15]. This may or may not be accompanied by radiotherapy and chemotherapy [5]. Several factors play a role in determining the prognosis of the case; histologic grade, reflected in the grade of differentiation, remains the most important prognostic factor. Low-grade myxoid variants have a five-year survival rate of 90%, while high-grade lipomatous tumours such as round cell and PLSs have survival rates of 60% and 30-50% [5]. Distant metastasis also plays a major role in the prognostics of the patient. While low-grade liposarcomas rarely metastasize, PLS, especially those in the deep-seated tissues of the lower extremities, tend to show early metastasis to the lungs in 75% of cases [5]. Bone metastases, on the other hand, is rarer in soft tissue sarcomas and varies significantly among histological subtypes; PLS, leiomyosarcoma, and angiosarcoma showed the highest incidence of bone metastasis, where axial skeleton is affected in 70% of the cases [5,6,13-15]. However, as displayed in this case, liver metastasis is extremely rare as medical literature has only reported two cases of isolated liver metastasis in PLS [5,6]. In most soft tissue sarcomas, adjuvant chemotherapy following surgery has been proved to be of dubious value; however, in high-grade sarcomas with tumour size > 5cm and widespread metastasis, chemotherapy use has been justified even though it is associated with complications such as delayed wound healing [5].

## Conclusions

PLS has the highest mortality rate among the four subtypes of liposarcomas, highlighting the importance of early and accurate diagnosis. Although IHC can be used in excluding other diagnoses, careful microscopic examination of the lipoblasts demonstrates that lipoblasts are indeed “in the eye of the beholder”. In terms of management, low-grade liposarcomas (well-differentiated and myxoid) can be largely managed with surgery alone provided the surgery is carefully planned and adequate clean margins are achieved. However, high-grade liposarcomas (round cell and pleomorphic), especially in extremities need to often be managed with neoadjuvant/adjuvant chemotherapy and radiation. Metastasis remains a considerable concern in high-grade liposarcomas, however, the paucity of literature in the area shows the need for more extensive research to develop more standardized guidelines for management as such malignancies mostly have poor outcomes.
